# A reexamination of krill oil bioavailability studies

**DOI:** 10.1186/1476-511X-13-137

**Published:** 2014-08-26

**Authors:** Norman Salem, Connye N Kuratko

**Affiliations:** Nutritional Lipids, DSM Nutritional Products, 6480 Dobbin Road, Columbia, MD 21045 USA

**Keywords:** Fish oil, Krill oil, Bioavailability, Omega-3 fatty acids

## Abstract

It has proven difficult to compare the bioavailability of krill oil (KO) vs. fish oil (FO) due to several of the characteristics of KO. These include the lower concentration of the active ingredients, eicosapentaenoic acid (EPA, 20:5n-3) and docosahexaenoic acid (DHA, 22:6n3), in KO as well as differences in their ratio relative to FO as well as the red color due to astaxanthin. In addition, the lipid classes in which EPA and DHA are found are quite different with KO containing phospholipid, di- and tri-glycerides as well as non-esterified fatty acid forms and with FO being primarily triglycerides. No human study has yet been performed that matches the dose of EPA and DHA in a randomized, controlled trial with measures of bloodstream EPA and DHA content. However, several claims have been made suggesting greater bioavailability of KO vs. FO. These have largely been based on a statistical argument where a somewhat lower dose of KO has been used to result in a similar bloodstream level of EPA and/or DHA or their total. However, the magnitude of the dosage differential is shown to be too small to be expected to result in differing blood levels of the long chain n-3 PUFAs. Some studies which have claimed to provide equal doses of KO and FO have actually used differing amounts of the two major n-3 fatty acid constituents. It is concluded that there is at present no evidence for greater bioavailability of KO vs. FO and that more carefully controlled human trials must be performed to establish their relative efficacies after chronic administration.

## Introduction

Modern diets particularly in the Western world are largely insufficient in the long chain omega-3 fatty acids eicosapentaenoic acid (EPA) and docosahexaenoic acid (DHA) [1] leading to sub-optimal organ function and an increased risk of disease [2]. This has led many expert bodies to make recommendations for increasing intakes of these vital nutrients either from an increased seafood intake or via supplements. However, seafood intake in the Western world is low and as a result EPA and DHA intake in the US, for example, is only about 30 and 60 mg/d, respectively, on average [3]. Thus, in order to increase intake of these nutrients, either enriched foods or high quality supplements containing EPA and DHA would be needed by many consumers. Industry has provided fish oils (FO), algal oils and krill oil (KO) capsules to fulfill this requirement. The EPA and DHA delivered in fish oil and algal oil is largely in the form of triglycerides whereas krill oil has triglyceride but also phospholipid and non-esterified fatty acid forms of these nutrients. Recent studies such as the one presented by Ramprasath et al. [4] have claimed that EPA and DHA in krill oil are more bioavailable or more efficiently absorbed than EPA/DHA in fish oils, presumably due to the phospholipid form of the lipids in KO. A greater bioavailability would translate into a higher tissue content of EPA and DHA and a resulting greater health benefit. However, it is contended here that this has not in fact been demonstrated due to study design limitations in this and other previous studies, as will be discussed below.

## Discussion

Ramprasath et al. [4] state in their original paper that “none of the studies managed to show improvement in the absorption of n-3 fatty acids with krill oil over fish oil”. They criticize the absence of a randomized, controlled trial design and the use of single dose or short term supplementation periods. Clinical studies which are double blinded, randomized and controlled prevent the introduction of biases. It is also not valid to interpret differing absorption characteristics of a single dose or a short term supplementation as relevant to the efficacy of various supplements which are to be given chronically. Several of the existing human studies where KO was compared to FO for its ability to change bloodstream fatty acid profiles used such approaches. Faced with the lower content of EPA and DHA in KO relative to FO, researchers did not attempt to provide equivalent fatty acid doses. Rather they provided less EPA/DHA in the KO capsules relative to the FO capsules and then argued that the KO was more bioavailable when the results showed similar blood levels of EPA and DHA. Table 1 provides a summary of key features of several of these human studies.

**Table 1:** **Study characteristics of comparisons of krill oil and fish oil induced bloodstream fatty acid compositional changes in humans**

Reference	Krill oil		Fish oil		Outcome	Adverse events	Study limitations
	EPA (mg/d)	DHA (mg/d)	EPA (mg/d)	DHA (mg/d)			
Laidlaw et al. [9]	150	90	As rTG: 650	As rTG: 450	Percent increase in whole blood fatty acids	No serious AEs	Open-label crossover study
			As EE: 756	As EE: 228	Extrapolation of data for blood composition at 1 g fatty acid intake	Other AEs not specified	EPA/DHA doses varied according to the lipid source
			As TG: 180	As TG: 220	Estimation of cardiovascular risk reduction		Short period of supplementation (4 wk)
Maki et al. [6]	216	90	212	176	4 wk supplementation with KO, menhaden oil, or olive oil	No safety-related AEs	Single dosage of EPA + DHA
					Change in total plasma fatty acid (μmol/L) from baseline	Frequency of gas/bloating and flatulence was greater in KO group than in FO group	Short period of supplementation (4 wk)
Ramprasath et al. [3, 9]	493 (calculated)	284 (calculated)	404 (calculated)	260 (calculated)	4 wk supplementation with KO, FO, or corn oil	No safety-related AEs	LA content was higher in FO supplement than in KO supplement
					Change in total plasma and RBC fatty acid (% TFA) from baseline	Burping and aftertaste was greater in KO group than in FO group	Short period of supplementation (4 wk)
							Single dosage of EPA + DHA
Schuchardt et al. [7]	1050	630	As TG: 1008	As TG: 672	Plasma PL fatty acid at baseline, 2, 4, 6, 8, 24, 48, and 72 h after the one-day intervention	NR	Acute supplementation trial
			As EE: 1008	As EE: 672	Comparisons made as change from baseline, area under the curve, C-max, and t-max		Crossover design with high standard deviations
Ulven et al. [4]	348	195	450	414	7 wk supplementation with KO, FO, or no supplementation	No safety-related AEs	Intervention was not blinded
					Change in total plasma fatty acid (μmol/L) from baseline	Difference in tolerability not reported	

EPA = eicosapentaenoic acid; DHA = docosahexaenoic acid; KO = krill oil; FO = fish oil; LA = linoleic acid (18:2n-6); %TFA = percent total fatty acid; RBC = red blood cell; EE = ethyl ester; rTG = re-esterified triglyceride; TG = triglyceride; AE = adverse event; NR, not reported.

One example of this is given by the study of Ulven et al. [5] where three commercial FO capsules containing a total of 864 mg of EPA/DHA were compared to six KO capsules containing a total of 543 mg EPA/DHA over a period of 7 wks; a control group was unsupplemented. Although the subjects, n = 36/group, were randomly assigned, they were not blinded as the three study groups were given capsules differing in number and appearance or no capsules for the control group. Groups taking more capsules may be expected to suffer from lower compliance and biases may be introduced in groups taking a capsule of a different color. Not only were the dosages quite different but the ratio of EPA/DHA was different; it was 1.12 in the FO and 1.74 in the KO. Plasma total lipids then showed a significantly increased but equal level of EPA and DHA as well as docosapentaenoic acid (DPAn-3) in the KO and FO groups. It is interesting to note that the KO group exhibited a significant 12% increase in arachidonic acid (ARA) whereas the FO group exhibited a 14% decrease. ARA and its eicosanoid products are important biomediators including their modulation of thrombotic effects and a lowering of ARA in adults is considered beneficial [6]. The authors conclude that “these findings indicate that the bioavailability of n-3 PUFAs from krill oil (mainly PL) is as, or possibly more, efficient as n-3 PUFA from fish oil (TG)”.

Similarly Maki et al. [7] in a randomized, controlled study of 76 obese adults given 2 g/d of KO, FO or olive oil for 4 wks provided similar amounts of EPA (216 vs. 212 mg/d) but more DHA in the FO group (90 vs. 178 mg/d). Again the plasma EPA and DHA were significantly increased in both the KO and FO groups relative to an olive oil control group but the two supplemented groups were the same from a statistical standpoint. It should be noted however that the increased DHA provided in the FO did result in a 66% greater increase in plasma DHA from the baseline value (149.9 vs. 90.2) relative to the KO group. The baseline values for EPA and particularly for DHA were much higher in the KO group than control suggesting that there were differences in diet or supplement use in that group relative to other subjects in the study. It is important in this type of study to exclude those subjects who take omega-3 supplements or eat large amounts of fish. It is also a good practice to collect food records or conduct food surveys of the patients in the study to assess omega-3 PUFA intake. This, together with the variability in the study obviated the attainment of statistically significant differences between groups.

In an acute study where KO was compared to a FO over 72 h in a cross-over trial in 12 healthy, young men, no statistically significant differences were observed in EPA, DHA or the sum of EPA + DHA in plasma phospholipids after giving a closely matched amount (1680 mg) of EPA/DHA [8]. There was only a 14 d wash out between the various legs of this trial and it is questionable whether this period is adequate for wash out. Again, although the authors indicate a double-blinded trial, they provided 14 capsules for the KO and only 4 capsules for the FO or FO ethyl ester arms. The (probable) difference in color as well as the different number of capsules preclude the possibility of a double-blinded study, potentially introducing bias.

There has been a very recent publication evaluating the efficacy of various sources of long chain omega-3 PUFAs when given at the doses recommended by their manufacturer [9]. This was an open label, randomized, cross-over study of 35 healthy subjects. The following sources were compared with the indicated EPA and DHA contents: concentrated triglyceride EPA 650, DHA 450 mg; fish oil ethyl ester EPA 756, DHA 228 mg; krill oil phospholipid EPA 150, DHA 90 mg; salmon oil triglyceride EPA 180, DHA 220 mg. As may be expected, the sources for which larger doses of EPA and DHA were delivered led to a higher content of EPA and DHA in whole blood after a 28 day exposure to the various supplements. For example, the whole blood EPA level was four-fold higher in the subjects given a concentrated triglyceride form of fish oil relative to the KO group. The authors also comment that various risk factors for cardiovascular disease were reduced best by this concentrated form of omega-3 PUFA but the less concentrated forms like the KO were relatively unsuccessful. Some effort was made to extrapolate the data to a standardized dosage of 1000 mg of EPA + DHA/d but the authors indicate that “because of the difficulties encountered in data extrapolation, i.e. the inaccuracies inherent in the methodology, given the negative results for some subjects, a statistical analysis of this data was not considered to be warranted”. They go on to conclude that “”a head-to-head comparison of the supplements utilized in this trial, at equivalent doses of EPA + DHA, would be useful in determining their relative bioavailability and their efficacy in increasing blood levels of omega-3 fatty acids, and in reducing CVD risk”. In another interesting note, these authors point out that some individuals actually had a decrease in blood EPA and DHA during supplementation indicating that they were not consuming their capsules. Thus the monitoring of compliance by interview, by capsule counts and via fatty acid analysis should always be a feature of these types of clinical trials. The authors suggest that one explanation for the decline in EPA and DHA content in some of these subjects may be due to their habitual intake of high amounts of these nutrients thru seafood in their diets. Once this trial began, they were asked to stop this seafood intake and the amount of EPA and DHA in the supplement was actually less than their previous intake, thus leading to a decline in their blood levels. Here again, it would be important to exclude those subjects consuming large amounts of EPA and DHA thru diet or via supplements.

A recent animal study has also made the claim of greater KO bioavailability relative to FO based on providing a lower dose and then finding a statistically similar plasma and liver content of EPA and DHA [10]. In fact, there was again a higher mean content of EPA in both tissues in the FO group with a substantial difference in liver but the differences with respect to KO did not reach statistical significance as there was considerable variability in measures and a small number of animals in these experimental groups, an n = 6.

Ramprasath et al. [4] recognized that previous studies failed to show differences in bioavailability or sustained changes in tissue fatty acid composition after chronic exposure to FO or KO. They performed a randomized, cross-over trial in 24 healthy young adults over a period of 4 wks. They purported to study the same dose of the two preparations and thus confronted the problem of the higher FO content of EPA and DHA by blending the FO with corn oil [11]. Their claim of performing a trial with matched omega-3 oils is questionable since, as pointed out by Nichols et al. the KO contained 114 mg greater EPA + DHA than the FO dose [12]. According to the data presented by Ramprasath in their Table six, the FO contained 13.5% of the fatty acids as EPA as compared to 16.4% in KO and 8.7% DHA as compared to 9.5% in the KO [4]. This translates, assuming that the same total amount of oil is given in each supplement, to an increase of 22% (16.44/13.46) in the EPA and 9.5% (9.46/8.66) in the DHA in the KO capsules relative to the FO capsules. That the DPAn-3 was slightly higher in the FO is not an equalizer since the main parameters to be compared were EPA and DHA as well as their sum in plasma and RBC. It should be apparent then that the doses of EPA/DHA were not the same between study arms.

In summary, the argument put forth in many of the KO studies is that if a lower dose of KO is given relative to that in the FO, and if this results in statistically the same EPA or DHA content in the bloodstream, the KO must have been more bioavailable or more efficacious. This seems to make sense to the non-scientist but is in fact based on a statistical argument. The argument makes the assumption that the differences in EPA/DHA dosage are of a magnitude such that they would be expected to result in a statistically significant difference in mean EPA, DHA or EPA + DHA content in plasma or other blood compartment. However, it is quite common that even very substantial differences in omega-3 dosing, although it may indeed lead to a quite different mean value for EPA or DHA, do not produce a statistically significant difference. Some examples of this are in order. In dose-response studies of FO supplementation, for example, we can compare various doses given within the same study to see how much of an increase in dose is required before an increased mean value of plasma EPA or DHA becomes statistically significant in similarly sized studies.

Barcelo-Coblijn et al. conducted a study of 62 firefighters divided into six experimental groups where the time course of n-3 fatty acid content in RBC was measured biweekly over 12 wks at a daily FO dose of either 0.6 or 1.2 g/d [13]. The total RBC n-3 fatty acid content was about the same between these two groups after 2 wks and began to diverge between 4-8 wks only to converge at the same mean value after 10 wks. Although there was a two-fold difference in FO dose, the difference in mean values at the 4 wk time point, for example, was only approximately 7%.

An even more instructive finding was obtained by Flock et al. in a dose-response study of 300, 600, 900 and 1800 mg/d of FO given daily to 115 healthy men for 5 months with measurements of RBC fatty acid content [14]. Their data for RBC EPA and DHA content is presented in Table 2. Inspection of this data demonstrates that if 300 and 600 mg/d FO doses are compared, there is an increase in the mean values for RBC EPA and DHA but these changes are not significantly different. The content of EPA and DHA increase regularly with dose consumed but it is apparent that there is not a linear increase. Note for example the much greater increase in RBC DHA when subjects are given the first 300 mg increment (1.43) than the second such increment (0.30). When the RBC EPA and DHA contents for the 600 and 900 mg/d doses are compared, there is again no statistical significance to the change in mean values. In this study, differences in EPA + DHA dosages of 600 mg/d were necessary to lead to statistically significant changes in mean values for EPA or DHA, i.e., statistical significance was reached for example between 300 and 900 mg/d of FO. This is not to indicate that smaller differences in doses cannot lead to significant changes in bloodstream EPA or DHA content in a particular trial. The contention is that the statistical significance of mean differences will depend upon the supplement dose, length of treatment, number of subjects in the groups, compliance with study protocols, control of diets and the variability of analytical measurements. It also points to the conclusion that the most reasonable design for a study comparing two sources of EPA and DHA differing in lipid class is one where identical amounts of both EPA and DHA are given. If one of the sources then produced a statistically different content of EPA or DHA, then a valid claim could be made concerning efficacy or bioavailability. It is important though to note that the differences in the KO vs. FO trials are less than 600 mg and so it is doubtful that one could expect a difference in EPA or DHA content. Therefore, the fact that the final composition of EPA, DHA or EPA + DHA was statistically speaking the same even though in many cases less KO dose was given, was to be expected and has no implications for bioavailability. Trials based on this premise cannot conclude that KO is more bioavailable than FO.

**Table 2:** **Effect of multiple doses of fish oil on human erythrocyte EPA and DHA content after 5 months of supplementation**

EPA + DHA dose (mg/d)	0	300	600	900	1800
**RBC EPA**	0.47 ± 0.09^a^	0.91 ± 0.09^b^	1.23 ± 0.10^bc^	1.44 ± 0.09^c^	2.46 ± 0.09^d^
**RBC DHA**	3.87 ± 0.16^a^	5.30 ± 0.16^b^	5.60 ± 0.17^bc^	6.06 ± 0.16^c^	7.03 ± 0.16^d^

Doses which produced a statistically different fatty acid content were denoted by differing superscripts (data from Flock et al. ref 12).

Nichols et al. [12] suggested that Ramprasath et al. [4] had introduced an inappropriate variable into their study as the FO capsules contained over 32% of their fatty acids as linoleic acid (18:2n-6, LA). In a response, Ramprasath et al. [11] indicated that they had blended the FO with corn oil in an attempt to equalize the levels of EPA and DHA in the two oils and conceded that this may have influenced their results. If LA was 32.5% of the fatty acid and 3 g of oil were given per day as their publication indicates, this would provide about 975 mg of LA per day in the FO group (only 62 mg in the KO group). The introduction of this variable is of importance as it is well known that there is an antagonistic effect of raising LA on EPA and DHA content in mammalian tissues [2, 6, 15]. A simple calculation could then be made using the empirical equations developed by Lands to estimate how much this factor lowered the values for total n-3 PUFAs in the FO case [15]. What is required for this calculation is the LA, ALA, n-6 HUFA and n-3 HUFA intake values. Since Ramprasath et al. performed their study in Canada, EFA intake values available for Canadian women [16] were used in this calculation. The calculation result is that adding an additional 913 mg of LA in the FO supplement, or about 0.365 en% LA, produces a 1% decrease in the n-3 expressed as a percentage of total HUFA (highly unsaturated fatty acids). This factor must then be applied to the values given by Ramprasath in their Tables two and three for the “Total n-3 PUFA” in the FO group at the end-point of the experiment [4]. Applying this correction to these fatty acid values indicates that the FO values for total n-3 PUFA would have been 6.69% (vs. 6.51%) in plasma and 8.30% (vs. 8.04%) in erythrocytes had not the additional LA been added to the FO capsules. Thus about 26 and 37% of the differences observed in plasma and erythrocytes, respectively, between KO and FO could be ascribed to this addition of linoleic acid into the FO. It is unclear whether this would have resulted in a lack of a statistical difference in the EPA and DHA content between the FO and KO groups but it would have made observing such a difference less likely.

It has been surmised that KO may be better absorbed relative to FO as it is partly comprised of phospholipid whereas FO products are mainly triglycerides. In fact, commercial KO compositions can vary widely in phospholipid content from as little as 19% up to 81% [17]. KO di- and tri-glycerides can vary from about 13 to 34% and non-esterified fatty acids can vary from 3.5 to as much as 36%. A report of a third commercial KO source indicates that it is composed of only 16% phospholipid, 24% triglyceride and 60% of a “polar, non-phospholipid fraction [18] which is composed mainly of cholesterol, mono- and diglycerides and red pigment, mainly astaxanthin” [19]. Thus two of the three commercial KO preparations had phospholipids as a minor component. Some support can be found in the literature for an increased efficacy for a phospholipid source of PUFAs relative to triglycerides. In a single dosing study, Wijendran et al found a better incorporation of a stable isotope labeled phospholipid containing labeled ARA relative to a triglyceride form [20]. However, such single dose studies cannot be extrapolated to a chronic case where supplements are taken daily and it is unknown whether DHA incorporation into tissues would have a similar sensitivity to the lipid form provided. Only the equilibrium values of these essential fatty acids in tissues are of relevance and clearly related to health effects.

In some of the KO publications, it is stated that there were no adverse effects observed but examination of the data in the papers indicate otherwise. For example, in the Ramprasath study, Table five indicates that subjects in both the corn oil placebo and fish oil groups had one incidence each of burping at a mild and moderate level of intensity [4]. However, there were 4 and 3 incidences at the mild/moderate levels of burping in the KO arm. Similarly, aftertaste was noted as mild on three occasions in the FO arm but 6 times in the KO arm and one at the more intense level of moderate. No statistical analyses appear to have been performed and yet the abstract concludes that “krill oil was well tolerated with no adverse effects”. It is clear that these are not serious adverse effects but they may well indicate sensory properties that are not up to the standard of commercial FO. Similarly, Maki et al. [7] indicate that the incidence of various gastrointestinal side effects in comparison to menhaden oil are increased, e.g., gas/bloating, 5 vs 0; flatulence, 9 vs 2; diarrhea, 5 vs 2. The authors indicate that the increased incidence of gas/bloating and flatulence in the KO group over that observed in the fish oil group was statistically significant. In spite of these observations, the authors state in the abstract that “No significant differences for other safety variables were noted, including adverse events”. Here again, these are not serious adverse events but they are indeed adverse events that can affect the quality of life when a supplement is taken chronically.

There has been a recent animal study that has raised some concern about possible KO kidney toxicity [18]. In this study KO was compared to several fish oils (menhaden (MO), salmon (SO) and tuna (TO)) as well as a flaxseed oil (FO) and a corn oil (CO) control after feeding to 4 wk old rats for a subsequent 8 wks as a 12% fat diet. The authors summarized their findings by saying “Rats fed MO, TO and SO had higher renal DHA and EPA content that may optimize health by reduced inflammation through decreasing production of mediators of inflammation, activation of transcription factors, and inflammatory gene expression. In contrast, rats consuming KO and to a lesser degree FO showed evidence of renal calcification and tubulo-interstitial injury. This was due to increased urinary P excretion associated with the phospholipids content of these oil sources. Although further studies are needed, susceptible individual should be aware of a potential risk of increasing phospholipids consumption on renal health”. (It is important to state here that within this quotation, the FO is an abbreviation for flaxseed oil and not fish oil). These results were obtained using a diet with 11.8 wt% as KO and this is a dose that is unlikely to occur in human diets. Nevertheless, as the authors point out, “use of high doses of purified compounds is standard when assessing the safety of compounds”.

In summary, a careful randomized, controlled trial is needed in healthy adults to compare triglyceride forms of long chain, omega-3 PUFAs as found in fish oils to the phospholipid form found in KO. It is necessary to adjust the FO EPA and DHA content to be equivalent to the KO and to blend them to the same concentration as the KO so that capsules could be made that match in size and dosage. Furthermore, FO capsules should be colored so that they are similar to the color of KO capsules. A chronic study would then be carried out for at least 4 wks and the primary endpoint being RBC or plasma fatty acid content of EPA and DHA at the study termination. Control over diet must be exerted to limit fish intake during the trial and subjects with prior omega-3 supplement use or high fish intake must be excluded. Statistical analyses would then be made to determine whether there are differences in EPA/DHA content when an identical dose is given in the two different forms.

## Abbreviations

FO: Fish oil
KO: Krill oil
EPA: Eicosapentaenoic acid, 20:5n-3
DPA: Docosapentaenoic acid, 22:5n-3
DHA: Docosahexaenoic acid, 22:6n-3
ARA: Arachidonic acid, 20:4n-6
EFA: Essential fatty acid
HUFA: Highly unsaturated fatty acids with ≥20C and ≥3 double bonds
PUFA: Polyunsaturated fatty acid.
